# *TCF7L2* Polymorphism, Weight Loss and Proinsulin∶Insulin Ratio in the Diabetes Prevention Program

**DOI:** 10.1371/journal.pone.0021518

**Published:** 2011-07-26

**Authors:** Jeanne M. McCaffery, Kathleen A. Jablonski, Paul W. Franks, Sam Dagogo-Jack, Rena R. Wing, William C. Knowler, Linda Delahanty, Dana Dabelea, Richard Hamman, Alan R. Shuldiner, Jose C. Florez, for the Diabetes Prevention Program Research Group

**Affiliations:** 1 The Biostatistics Center, The George Washington University, Rockville, Maryland, United States of America; 2 Weight Control and Diabetes Research Center, The Miriam Hospital and Brown Medical School, Providence, Rhode Island, United States of America; 3 Department of Genetic and Molecular Epidemiology, Lund University, Malmo, Sweden; 4 Division of Endocrinology, Diabetes and Metabolism, University of Tennessee College of Medicine, Memphis, Tennessee, United States of America; 5 Diabetes Epidemiology and Clinical Research Section, National Institute of Diabetes and Digestive and Kidney Diseases, Phoenix, Arizona, United States of America; 6 Massachusetts General Hospital Diabetes Center, Boston, Massachusetts, United States of America; 7 Department of Epidemiology, Colorado School of Public Health, Aurora, Colorado, United States of America; 8 Division of Endocrinology, Diabetes and Nutrition, University of Maryland School of Medicine, Baltimore, Maryland, United States of America; 9 Geriatrics Research and Education Clinical Center, Veterans Administration Medical Center, Baltimore, Maryland, United States of America; 10 Diabetes Research Center Diabetes Unit and Center for Human Genetic Research, Massachusetts General Hospital, Boston, Massachusetts, United States of America; 11 Department of Medicine, Harvard Medical School, Boston, Massachusetts, United States of America; 12 Program in Medical and Population Genetics, Broad Institute, Cambridge, Massachusetts, United States of America; University of Las Palmas de Gran Canaria, Spain

## Abstract

**Aims:**

*TCF7L2* variants have been associated with type 2 diabetes, body mass index (BMI), and deficits in proinsulin processing and insulin secretion. Here we sought to test whether these effects were apparent in high-risk individuals and modify treatment responses.

**Methods:**

We examined the potential role of the *TCF7L2* rs7903146 variant in predicting resistance to weight loss or a lack of improvement of proinsulin processing during 2.5-years of follow-up participants (N = 2,994) from the Diabetes Prevention Program (DPP), a randomized controlled trial designed to prevent or delay diabetes in high-risk adults.

**Results:**

We observed no difference in the degree of weight loss by rs7903146 genotypes. However, the T allele (conferring higher risk of diabetes) at rs7903146 was associated with higher fasting proinsulin at baseline (*P*<0.001), higher baseline proinsulin∶insulin ratio (p<0.0001) and increased proinsulin∶insulin ratio over a median of 2.5 years of follow-up (*P* = 0.003). Effects were comparable across treatment arms.

**Conclusions:**

The combination of a lack of impact of the *TCF7L2* genotypes on the ability to lose weight, but the presence of a consistent effect on the proinsulin∶insulin ratio over the course of DPP, suggests that high-risk genotype carriers at this locus can successfully lose weight to counter diabetes risk despite persistent deficits in insulin production.

## Introduction

It is well established that the rs7903146 variant in the transcription factor 7-like 2 gene (*TCF7L2*) is associated with type 2 diabetes (T2D) [1]–[4]. The TT genotype at rs7903146 also predicted progression to diabetes in the Diabetes Prevention Program (DPP) [5] and the Finnish Diabetes Prevention Study [6].

The extent to which rs7903146 may also be associated with body mass index (BMI) and ability to lose weight remains controversial. Helgason and colleagues [7] reported that a haplotype including the C allele at rs7903146 and the A allele at rs10885406 (HapA) was positively associated with BMI, whereas the absence of HapA (noted as HapB) including the diabetes risk T allele at rs7903146 was negatively associated with BMI. The negative association between the diabetes risk T allele at rs7903146 and BMI was also observed in DPP at baseline [5]. However, no direct effect of the T allele on BMI has been seen in population-based samples [8], [9].

Two studies that have evaluated whether rs7903146 alters weight loss response to behavioral intervention have had differing outcomes. Cauchi *et al.* documented a lack of impact of rs7903146 on the extent of weight loss in response to a 10-week hypocaloric diet [8]. However, Haupt et al. found that carriers of the T allele at rs7903146 lost less weight in response to an exercise and dietary intervention than CC homozygotes [10], suggesting a potential link between *TCF7L2* and resistance to weight loss.

In the DPP, the diabetes risk T allele at rs7903146 was also associated with impaired insulin secretion [5]. Despite active research on the mechanism by which variation in *TCF7L2* increases diabetes risk and affects β-cell function, the precise mode of action is poorly understood. One hypothesis involves interference with proinsulin processing. Loos and colleagues reported a highly significant association of the T allele at rs7903146 with circulating fasting proinsulin and the proinsulin∶insulin ratio in healthy white adults from the United Kingdom [11]. This association has since been replicated [9], [12], [13], and recent animal experimentation suggests that *TCF7L2* silencing may impair insulin vesicle trafficking [14]. The extent to which rs7903146 may alter the beneficial impact of lifestyle intervention and metformin on proinsulin∶insulin ratio remains to be determined.

In the present paper, we sought to examine whether rs7903146 and HapA in *TCF7L2* are also associated with: 1) resistance to weight loss; and 2) a lack of improvement in proinsulin∶insulin ratio in over a median of 2.5 year follow-up in DPP.

## Materials and Methods

### Ethics Statement

The Office of Human Research at The George Washington University Medical Center is the institutional review board responsible for insuring that The Biostatistics Center adheres to the principles expressed in the Declaration of Helsinki in approving the coordination, research, and analysis of data for the Diabetes Prevention Program (DPP) and Diabetes Prevention Program Outcomes Study (DPPOS). The participants of the DPP /DPPOS were not placed at undue risk and have provided written informed consent for their participation in all aspects of the study protocol. The consents obtained from participants at individual DPP clinics were approved by that clinic's institutional review board.

### Sample

The DPP [15], [16] enrolled 3,234 participants at high risk of developing diabetes (on the basis of increased fasting glucose, impaired glucose tolerance, and a BMI of 24 or higher) across 27 clinical centers throughout the U.S. Participants were randomized to placebo, metformin 850 mg twice daily or a lifestyle intervention aiming at ≥7% weight loss and ≥150 minutes of physical activity per week. The average weight loss was 0.1, 2.1 and 5.6 kg in the placebo, metformin and lifestyle interventions, respectively, during a median of 2.5 years follow-up (median = 2.5 years, range 0.5–5.0 years). The 2,994 participants presented in this paper provided informed consent specifically for genetic analyses.

### Height and Weight

Standing height was determined in duplicate at baseline using a calibrated stadiometer. Weight was measured in duplicate on a calibrated balance beam scale semi-annually.

### Proinsulin and insulin

Fasting blood was drawn annually for measurements of insulin and proinsulin. Insulin measurements were performed using a radioimmunoassay method incorporating an anti–guinea pig antibody that measures total immunoreactive insulin. The assay is a 48-h polyethylene glycol–accelerated method with coefficients of variation (CVs.) of 4.5% for high-concentration quality control samples and 6.9% for low-concentration quality control samples. The CV for masked split duplicates in this assay was 8.5%. Proinsulin was measured by a commercially available radioimmunoassay method (Linco Research, St. Louis, MO) [15].

### Genotyping

Genotyping was performed by allele-specific primer extension of multiplex amplified products and detection using matrix-assisted laser desorption ionization-time-of-flight mass spectrometry on a Sequenom iPLEX platform [17] as previously described [5]. The rs7903146, rs10885406 and rs7924080 SNPs were selected based on prior association with diabetes and/or BMI [4], [5]. Hardy-Weinberg equilibrium was observed for the SNPs in all major race/ethnic groups.

As noted by Helgason *et al.*, [4] HapA can be tagged by the combination of the A allele at rs10885406 and the C allele at rs7903146, or by the T allele at rs7924080; for the purposes of this manuscript, we used the first definition. Haplotypes were determined within each self-reported ethnic group by using the expectation-maximization algorithm of Excoffier and Slatkin [18] modified to process larger data files using the partition-ligation approach. We confirmed that HapA defined by rs10885406 and rs7903146 is highly correlated with rs7924080 in our dataset, with r^2^ = 0.98 in whites, 0.97 in African Americans, 0.99 in Hispanics, 0.91 in Asians and 1.0 in Native Americans.

### Statistical analysis

The primary analyses involved testing whether weight, proinsulin∶insulin ratio, and the numerator and denominator of the ratio, proinsulin and insulin, differed by genotype at rs7903146 or HapA at baseline and over a median of 2.5 year follow-up. We used analyses of covariance with a genotypic model to test baseline differences in weight and natural log transformed proinsulin, insulin and proinsulin∶insulin ratio. Age, sex, ethnicity were statistically adjusted in tests for baseline weight. BMI and HOMA-IR, i.e., the product of fasting insulin and fasting glucose concentrations, were also statistically adjusted in baseline models for proinsulin, insulin and proinsulin∶insulin ratio.

General linear models for fixed effects were used to test the relationship between the *TCF7L2* variants and longitudinal change in weight, proinsulin, insulin and proinsulin∶insulin ratio over 2.5 years of follow-up. All longitudinal models were adjusted by age, sex, ethnicity, and the corresponding baseline parameter. Analyses of proinsulin, insulin and proinsulin∶insulin ratio were further adjusted for baseline BMI and fasting glucose. In each analysis a genotype×treatment arm interaction was included. Nominal two-sided *P* values are reported adjusted for multiple comparisons within each genotype using the Holm procedure [19]. All analyses were conducted in SAS, version 9.1 (SAS Institute, Inc, Cary, NC).

## Results

Demographic characteristics, baseline anthropometric characteristics and baseline proinsulin and insulin levels and proinsulin∶insulin ratios are presented in Table 1. No significant differences between treatment arms were observed at baseline. At follow-up, the treatment arms differed significantly in weight loss, proinsulin and insulin concentrations and proinsulin∶insulin ratio.

**Table 1:** Demographic characteristics and baseline and change in weight, proinsulin, insulin and proinsulin∶insulin ratio over 2.5 year follow-up.

	Treatment Group		
	Placebo	Metformin	Lifestyle
***Baseline***			
N	1000	990	1004
Male	311 (31.1%)	344 (34.7%)	327 (32.6%)
Age (yr)	50.5±10.5	51.0±10.4	50.7±11.4
Self-reported ethnicity			
Caucasian	557 (55.7%)	570 (57.6%)	547 (54.5%)
African American	210 (21.0%)	206 (20.8%)	196 (19.5%)
Hispanic	164 (16.4%)	158 (16.0%)	176 (17.5%)
Asian/Pacific Islander	39 ( 3.9%)	33 ( 3.3%)	56 ( 5.6%)
American Indian	30 ( 3.0%)	23 ( 2.3%)	29 ( 2.9%)
rs7903146			
C C	509 (51.2%)	447 (45.3%)	513 (51.2%)
C T	404 (40.6%)	447 (45.3%)	376 (37.5%)
T T	82 ( 8.2%)	92 ( 9.3%)	113 (11.3%)
HapA^a^			
PP	285 (28.5)	239 (24.1)	276 (27.5)
AP	441 (44.1)	451 (45.6)	413 (41.1)
AA	274 (27.4)	300 (30.3)	315 (31.4)
Weight (kg)	94.8±20.2	94.6±20.0	94.5±20.8
BMI (kg/m^2^)	34.3±6.7	34.0±6.7	34.0±6.8
Waist (cm)	105±14.5	105±14.5	105±14.9
Glucose (mg/dL)	107.0±8.4	106.7±8.4	106.5±7.9

	Median (min,max)	Median (min,max)	Median (min,max)
Proinsulin (pM)	14 (3 to 144)	14 (2 to 131)	14 (2 to 120)
Insulin (pmol/L)	167 (21 to 1153)	167 (21 to 722)	167 (17 to 1278)
Proinsulin/insulin ratio (%)	10 (2 to 95)	11 (1 to 97)	11 (2 to 101)

*Follow up* b	Mean (95% CI)	Mean (95% CI)	Mean (95% CI)	*P* val*
Change in Weight (kg)	0.95 (0.12–1.78)	−0.78 (−1.61–0.04)	−4.53 (−5.36–3.71)	<0.001
Change in Proinsulin (pM)	1.19 (0.52 to 1.86)	−2.76 (−3.43 to −2.08)	−3.30 (−3.96 to −2.64)	<0.001
Change in Insulin (pmol/L)	2.40 (1.53 to 3.28)	−1.63 (−2.52 to −0.737)	−2.17 (−3.04 to −1.30)	<0.001
Change in Insulin/Proinsulin Ratio (%)	−0.25 (−0.29 to 0.78)	−0.68 (−1.2 to −0.13)	−0.12 (−0.65 to 0.42)	<0.001

*P values from mixed models adjusted for baseline variable, age, self-reported ethnicity and sex.

a P - HapA present; A - HapA absent.

b Median follow-up 2.5 years.

Statistics shown are number of subjects and percents within each treatment group or means +/− standard deviation unless noted.

### Weight loss

As previously reported, the T allele at rs7903146 was associated with a lower BMI at baseline (*P*<0.01). No differences in baseline caloric intake or physical activity by genotype were observed (*P* = 0.70 and 0.23, respectively), suggesting that these measures did not account for the association of rs7903146 with baseline BMI. No association between HapA and BMI was seen in this cohort (*P* = 0.32).

**Figure 1:**
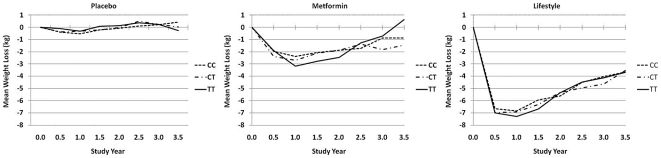
Mean weight loss in the Diabetes Prevention Program by treatment group and genotype at rs7903146.

Weight loss by rs7903146 is presented in Figure 1. There were no differences in the degree of weight loss by genotype at rs7903146 (*P* = 0.95) with no significant interaction of genotype with treatment arm (*P* = 0.82). Similarly, there were also no differences in the degree of weight loss by haplotype at HapA (*P* = 0.99), with no interaction of haplotype with treatment arm (*P* = 0.28).

### Proinsulin, insulin and proinsulin∶insulin ratio

Proinsulin, insulin and proinsulin∶insulin ratio at baseline and change in these parameters over a median of 2.5 year follow up are presented in Table 2. Genotypes at rs7903146 were not significantly associated with fasting proinsulin at baseline or over 2.5 year follow-up. No interactions with treatment arm were observed.

**Table 2:** Proinsulin, insulin and proinsulin∶insulin ratio at baseline and change in proinsulin, insulin and proinsulin∶insulin ratio over 2.5 year follow up by genotype at rs7903146.

	Baseline					Follow up (Change from Baseline)				
Variable	*P* valF-test	Genotype	LS Means*(95% CI)Back Transformed	Pair-WiseComparison	Adj*P* val**	*P* valF-test	Genotype	LS Means*(95% CI)	Pair-WiseComparison	Adj*P* val**
**Proinsulin (pM)**	0.805	CC	16.61 (15.99– 17.25)			0.262	CC	−0.434 (−1.197–0.329)		
		CT	16.78 (16.06– 17.53)				CT	0.084 (−0.790 – 0.958)		
		TT	16.95 (15.78– 18.21)				TT	0.535 (−0.888 – 1.959)		
**Insulin (pM)**	<0.001	CC	176.42 (170.55– 182.49)	CC vs CT	0.001	0.337	CC	−2.551 (−7.833 – 2.731)		
		CT	165.66 (159.33– 172.23)	CC, vs TT	0.001		CT	−6.803 (−12.825–−0.780)		
		TT	157.59 (147.83– 167.99)	CT, vs TT	0.129		TT	−4.537 (−14.398–5.324)		
**Proinsulin/Insulin Ratio**	<.001	CC	0.11 (0.11– 0.11)	CC vs CT	<0.001	<0.001	CC	−0.006 (−0.010–−0.001)	CC vs CT	0.054
		CT	0.12 (0.11– 0.12)	CC, vs TT	<0.001		CT	0.004 (−0.001–0.009)	CC, vs TT	0.268
		TT	0.12 (0.12– 0.13)	CT, vs TT	0.069		TT	0.002 (−0.007–0.010)	CT, vs TT	0.700

*Models adjusted for BMI, age, sex, ethnicity, and fasting glucose, follow-up values adjusted for the corresponding baseline.

**Holm's procedure for multiple comparisons.

The rs7903146 SNP was significantly associated with fasting insulin at baseline. The TT genotype at rs7903146 was associated with the lowest insulin level, differing significantly from CC genotypes. CT genotypes also exhibited lower fasting insulin than CC genotypes. Once baseline levels were statistically controlled, no differences in the change in insulin level by rs7903146 were observed over 2.5 year follow-up and no interactions with treatment arm were observed.

**Figure 2:**
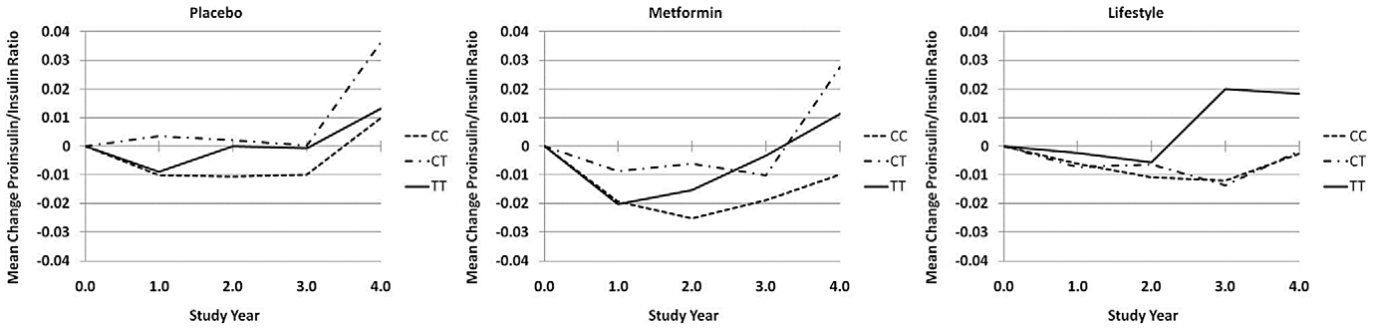
Mean change in proinsulin∶insulin ratio in the Diabetes Prevention Program by treatment group and genotype at rs7903146.

The rs7903146 SNP was also significantly associated with the proinsulin∶insulin ratio at baseline. At baseline, the TT genotype at rs7903146 was associated with the highest proinsulin∶insulin ratio, differing significantly from CC genotypes and tending to differ from CT genotypes. The CT genotype was also associated with a higher proinsulin∶insulin ratio than the CC genotype. Genotype at rs7903146 was also significantly associated with change in proinsulin∶ insulin ratio over the median of 2.5-year follow up once baseline levels were statistically controlled. Carriers of CC genotypes showed a decline in proinsulin∶insulin ratio across the 2.5-year follow-up relative to carriers of CT and TT genotypes who experienced an increase. No heterogeneity in these effects by treatment group was observed. Mean change in proinsulin∶insulin ratio over the course of the study by rs7903146 are presented in Figure 2.

The absence of HapA was significantly associated with lower insulin (*P* = 0.04) and greater proinsulin∶insulin ratio at baseline (*P* = 0.01) at baseline. The absence of HapA was also associated with a blunted decline in proinsulin∶insulin ratio over 2.5 year follow-up (*P* = 0.04). In order to distinguish whether the association signal was due to rs7903146 or HapA (which are highly correlated), we performed stratified analyses of the association of one variant with proinsulin∶insulin ratio within each genotypic group for the other. The only significant association in these stratified analyses was found for the T allele at rs7903146 within HapA heterozygotes (*P*<0.001), suggesting that the association is driven by rs7903146 and not HapA.

## Discussion

The results of this study indicate that the variation in the *TCF7L2* gene previously associated with T2D and BMI does not predict ability to lose weight. This effect was consistent across those randomized to the intensive lifestyle, metformin treatment, or placebo interventions, indicating that the extent and mode of weight loss do not influence the genetic associations. However, the T allele at rs7903146 was associated with higher proinsulin∶insulin ratio at baseline and, once baseline values were statistically adjusted for, change in the proinsulin∶insulin ratio over a median of 2.5 year follow-up. This effect was consistent across treatment arms in DPP despite significant weight losses in the lifestyle and metformin groups, relative to the placebo group.

As new genetic associations with T2D continue to emerge, it is plausible that genetic information may be used to guide the choice of preventive interventions. Before this can be achieved, it will be important to document the potential impact of these diabetes risk loci on the effectiveness of both pharmacological and behavioral interventions. For example, the extent of weight loss in response to a standardized caloric restriction regimen is highly heritable in twin studies [20]. Genetic loci previously associated with T2D or obesity serve as leading candidates that may promote resistance to weight loss in response to a behavioral intervention. In the current paper, we find that the diabetes risk marker rs7903146 in *TCF7L2* does not impact the ability to lose weight in response to metformin or behavioral intervention. This is consistent with one prior paper reporting no association of rs7903146 with the extent of weight loss in response to a short-term hypocaloric diet [8]. However, were unable to replicate a second paper from the Tuebingen Lifestyle Intervention Program (TULIP) reporting that carriers of the T allele showed blunted weight loss in response to a nine-month combined diet and exercise program [10]. Of relevance to this report, we found no heterogeneity of effect across treatment arms, indicating that the effect of genotype did not differ in the lifestyle intervention arm, relative to the metformin or placebo arms. We also found no heterogeneity by time, indicating that genotype effects at year 1 did not differ significantly from other time points. Several potential differences across the studies may account for the different results, including length of follow-up, nature or intensity of the lifestyle intervention and the extent of weight loss achieved. Of note, the TULIP included a dietary fiber intervention not included in the DPP. Taken together with a recent report indicating that dietary fiber may alter the association between *TCF7L2* and diabetes [21], it remains possible that changing certain components of the diet, such as fiber intake, may alter risks associated with *TCF7L2* variants.

This paper also extends prior results examining the impact of this locus on proinsulin∶insulin ratio. A greater proinsulin∶insulin ratio is thought to reflect a deficit in β-cell secretory function and *TCF7L2* appears to play a critical role in β-cell survival and glucose-stimulated insulin secretion [22]. Greater proinsulin∶insulin ratios have consistently been shown among T allele carriers at rs7903146 but associations with proinsulin level have been less consistent [9], [11]–[13]. In the present study, we found that rs7903146 is associated with a lower insulin level and, as a result, an elevated proinsulin∶insulin ratio at baseline. Further, the elevated proinsulin∶insulin ratio persisted over 2.5 years of follow-up once baseline values were statistically controlled for and was consistent across treatment arms. Thus, the impact of genotype on the proinsulin∶insulin ratio does not appear to be altered by metformin or lifestyle intervention. Proinsulin∶insulin ratio decreased in the metformin and lifestyle intervention arms in line with the expected improvement in proinsulin∶insulin ratio with increased insulin sensitivity. However, the persistence of differences in proinsulin∶insulin ratios across genotypic groups at *TCF7L2* rs7903146, despite interventions shown to significantly improve insulin sensitivity [23], raises the possibility that this defect may be caused by a specific effect of *TCF7L2* on proinsulin processing, β-cell proliferation and/or differentiation, or insulin vesicle trafficking, rather than by the general stress brought on the β cell by increased insulin demand.

Helgason and colleagues [7] reported that a haplotype including the C allele at rs7903146 and the A allele at rs10885406 (HapA) was positively associated with BMI, whereas the absence of HapA (noted as HapB) including the diabetes risk T allele at rs7903146 was negatively associated with BMI. We found the associations of HapA with weight and proinsulin∶insulin ratio to largely be driven by variation in the component SNP, rs7903146.

Taken together, the combination of a lack of impact of *TCF7L2* genotypes on the ability to lose weight in this study, but its consistent impact on the proinsulin∶insulin ratio, suggests that persons who carry high-risk genotypes at this locus can successfully lose weight to counter diabetes risk, despite consistent deficits in proinsulin processing.

## Supporting Information

Text S1 A complete list of DPP Centers, investigators, and staff.
(PDF)
