# Supplementary material for: TCF7L2 Polymorphism, Weight Loss and Proinsulin∶Insulin Ratio in the Diabetes Prevention Program
Source: PLoS One. 2011 Jul 26;6(7):e21518. doi: 10.1371/journal.pone.0021518 (PMC3144193; doi:10.1371/journal.pone.0021518)
Supplement: Text S1 — A complete list of DPP Centers, investigators, and staff. (PDF) [file pone.0021518.s001.pdf]

Text S1: DPP Research Group Investigators (to Aug 2002)
Updated- June 2011

Pennington Biomedical Research Center (Baton Rouge, LA)
George A. Bray, MD*
Iris W. Culbert, BSN, RN, CCRC**
Catherine M. Champagne, PhD, RD
Barbara Eberhardt, RD, LDN
Frank Greenway, MD
Fonda G. Guillory, LPN
April A. Herbert, RD
Michael L. Jeffirs, LPN
Betty M. Kennedy, MPA
Jennifer C. Lovejoy, PhD
Laura H. Morris, BS
Lee E. Melancon, BA, BS
Donna Ryan, MD
Deborah A. Sanford, LPN
Kenneth G. Smith, BS, MT
Lisa L. Smith, BS
Julia A. St.Amant, RTR
Richard T. Tulley, PhD
Paula C. Vicknair, MS, RD
Donald Williamson, PhD
Jeffery J. Zachwieja, PhD

***University of Chicago* (Chicago, IL)**
Kenneth S. Polonsky, MD*
Janet Tobian, MD, PhD*
David Ehrmann, MD*
Margaret J. Matulik, RN, BSN**
Bart Clark, MD
Kirsten Czech, MS
Catherine DeSandre, BA
Ruthanne Hilbrich, RD
Wylie McNabb, EdD
Ann R. Semenske, MS, RD

***Jefferson Medical College* (Philadelphia, PA)**
Jose F. Caro, MD*
Pamela G. Watson, RN, ScD*
Barry J. Goldstein, MD, PhD*
Kellie A. Smith, RN, MSN**
Jewel Mendoza, RN, BSN**
Renee Liberoni, MPH
Constance Pepe, MS, RD
John Spandorfer, MD

***University of Miami* (Miami, FL)**
Richard P. Donahue, PhD*
Ronald B. Goldberg, MD*
Ronald Prineas, MD, PhD*
Patricia Rowe, MPA**
Jeanette Calles, MSEd
Paul Cassanova-Romero, MD
Hermes J. Florez, MD
Anna Giannella, RD, MS
Lascelles Kirby, MS
Carmen Larreal
Valerie McLymont, RN
Jadell Mendez
Juliet Ojito, RN
Arlette Perry, PhD
Patrice Saab, PhD

***The University of Texas Health Science Center* (San Antonio, TX)**
Steven M. Haffner, MD, MPH*
Maria G. Montez, RN, MSHP, CDE**
Carlos Lorenzo, MD, PhD
Arlene Martinez, RN, BSN, CDE

***University of Colorado* (Denver, CO)**
Richard F. Hamman, MD, DrPH*
Patricia V. Nash, MS**
Lisa Testaverde, MS**
Denise R. Anderson, RN, BSN
Larry B. Ballonoff, MD
Alexis Bouffard, MA,
B. Ned Calonge, MD, MPH
Lynne Delve
Martha Farago, RN
James O. Hill, PhD
Shelley R. Hoyer, BS
Bonnie T. Jortberg, MS, RD, CDE
Dione Lenz, RN, BSN
Marsha Miller, MS, RD
David W. Price, MD
Judith G. Regensteiner, PhD
Helen Seagle, MS, RD
Carissa M. Smith, BS
Sheila C. Steinke, MS
Brent VanDorsten, PhD

***Joslin Diabetes Center* (Boston, MA)**
Edward S. Horton, MD*
Kathleen E. Lawton, RN**
Ronald A. Arky, MD
Marybeth Bryant
Jacqueline P. Burke, BSN
Enrique Caballero, MD
Karen M. Callaphan, BA
Om P. Ganda, MD
Therese Franklin
Sharon D. Jackson, MS, RD, CDE
Alan M. Jacobsen, MD
Lyn M. Kula, RD
Margaret Kocal, RN, CDE
Maureen A. Malloy, BS
Maryanne Nicosia, MS, RD
Cathryn F. Oldmixon, RN

* denotes Principal Investigator
** denotes Program Coordinator

Jocelyn Pan, BS, MPH
Marizel Quitingon
Stacy Rubtchinsky, BS
Ellen W. Seely, MD
Dana Schweizer, BSN
Donald Simonson, MD
Fannie Smith, MD
Caren G. Solomon, MD, MPH
James Warram, MD

***VA Puget Sound Health Care System and University of Washington* (Seattle, WA)**

Steven E. Kahn, MB, ChB*
Brenda K. Montgomery, RN, BSN, CDE**
Wilfred Fujimoto, MD
Robert H. Knopp, MD
Edward W. Lipkin, MD
Michelle Marr, BA
Dace Trence, MD

***University of Tennessee* (Memphis, TN)**

Abbas E. Kitabchi, PhD, MD, FACP*
Mary E. Murphy, RN, MS, CDE, MBA**
William B. Applegate, MD, MPH
Michael Bryer-Ash, MD
Sandra L. Frieson, RN
Raed Imseis, MD
Helen Lambeth, RN, BSN
Lynne C. Lichtermann, RN, BSN
Hooman Oktaei, MD
Lily M.K. Rutledge, RN, BSN
Amy R. Sherman, RD, LD
Clara M. Smith, RD, MHP, LDN
Judith E. Soberman, MD
Beverly Williams-Cleaves, MD

***Northwestern University's Feinberg School of Medicine* (Chicago, IL)**

Boyd E. Metzger, MD*
Mariana K. Johnson, MS, RN**
Catherine Behrends
Michelle Cook, MS
Marian Fitzgibbon, PhD
Mimi M. Giles, MS, RD
Deloris Heard, MA
Cheryl K.H. Johnson, MS, RN
Diane Larsen, BS
Anne Lowe, BS
Megan Lyman, BS
David McPherson, MD
Mark E. Molitch, MD
Thomas Pitts, MD
Renee Reinhart, RN, MS
Susan Roston, RN, RD
Pamela A. Schinleber, RN, MS

***Massachusetts General Hospital* (Boston, MA)**

David M. Nathan, MD*
Charles McKitrick, BSN**
Heather Turgeon, BSN**
Kathy Abbott
Ellen Anderson, MS, RD
Laurie Bissett, MS, RD
Enrico Cagliero, MD
Jose C. Florez, MD, PhD+
Linda Delahanty, MS, RD
Valerie Goldman, MS, RD
Alexandra Poulos

***University of California-San Diego* (San Diego, CA)**

Jerrold M. Olefsky, MD*
Mary Lou Carrion-Petersen, RN, BSN**
Elizabeth Barrett-Connor, MD
Steven V. Edelman, MD
Robert R. Henry, MD
Javiva Horne, RD
Simona Szerdi Janesch, BA
Diana Leos, RN, BSN
Sundar Mudaliar, MD
William Polonsky, PhD
Jean Smith, RN
Karen Vejvoda, RN, BSN, CDE, CCRC

***St. Luke's-Roosevelt Hospital* (New York, NY)**

F. Xavier Pi-Sunyer, MD*
Jane E. Lee, MS**
David B. Allison, PhD
Nancy J. Aronoff, MS, RD
Jill P. Crandall, MD
Sandra T. Foo, MD
Carmen Pal, MD
Kathy Parkes, RN
Mary Beth Pena, RN
Ellen S. Rooney, BA
Gretchen E.H. Van Wye, MA
Kristine A. Viscovich, ANP

***Indiana University* (Indianapolis, IN)**

David G. Marrero, PhD*
Melvin J. Prince, MD*
Susie M. Kelly, RN, CDE**
Yolanda F. Dotson, BS
Edwin S. Fineberg, MD
John C. Guare, PhD
Angela M. Hadden
James M. Ignaut, MA
Marcia L. Jackson
Marion S. Kirkman, MD

* denotes Principal Investigator
** denotes Program Coordinator

Kieren J. Mather, MD
Beverly D. Porter, MSN
Paris J. Roach, MD
Nancy D. Rowland, BS, MS
Madelyn L. Wheeler, RD

***Medstar Research Institute* (Washington, DC)**

Robert E. Ratner, MD*
Gretchen Youssef, RD, CDE**
Sue Shapiro, RN, BSN, CCRC**
Catherine Bavido-Arrage, MS, RD, LD
Geraldine Boggs, MSN, RN
Marjorie Bronsord, MS, RD, CDE
Ernestine Brown
Wayman W. Cheatham, MD
Susan Cola
Cindy Evans
Peggy Gibbs
Tracy Kellum, MS, RD, CDE
Claresa Levatan, MD
Asha K. Nair, BS
Maureen Passaro, MD
Gabriel Uwaifo, MD

***University of Southern California/UCLA Research Center* (Alhambra, CA)**

Mohammed F. Saad, MD*
Maria Budget**
Sujata Jinagouda, MD**
Khan Akbar, MD
Claudia Conzues
Perpetua Magpuri
Kathy Ngo
Amer Rassam, MD
Debra Waters
Kathy Xapthalamous

***Washington University* (St. Louis, MO)**

Julio V. Santiago, MD* (deceased)
Samuel Dagogo-Jack, MD, MSc, FRCP, FACP*
Neil H. White, MD, CDE*
Samia Das, MS, MBA, RD, LD**
Ana Santiago, RD**
Angela Brown, MD
Edwin Fisher, PhD
Emma Hurt, RN
Tracy Jones, RN
Michelle Kerr, RD
Lucy Ryder, RN
Cormarie Wernimont, MS, RD

***Johns Hopkins School of Medicine* (Baltimore, MD)**

Christopher D. Saudek, MD*
Vanessa Bradley, BA**
Emily Sullivan, MEd, RN**
Tracy Whittington, BS**
Caroline Abbas
Frederick L. Brancati, MD, MHS
Jeanne M. Clark, MD
Jeanne B. Charleston, RN, MSN
Janice Freel
Katherine Horak, RD
Dawn Jiggetts
Deloris Johnson
Hope Joseph
Kimberly Loman
Henry Mosley
Richard R. Rubin, PhD
Alafia Samuels, MD
Kerry J. Stewart, EdD
Paula Williamson

***University of New Mexico* (Albuquerque, NM)**

David S. Schade, MD*
Karwyn S. Adams, RN, MSN**
Carolyn Johannes, RN, CDE**
Leslie F. Atler, PhD
Patrick J. Boyle, MD
Mark R. Burge, MD
Janene L. Canady, RN, CDE
Lisa Chai, RN
Ysela Gonzales, RN, MSN
Doris A. Hernandez-McGinnis
Patricia Katz, LPN
Carolyn King
Amer Rassam, MD
Sofya Rubinchik, MD
Willette Senter, RD
Debra Waters, PhD

***Albert Einstein College of Medicine* (Bronx, NY)**

Harry Shamoon, MD*
Janet O. Brown, RN, MPH, MSN**
Elsie Adorno, BS
Liane Cox, MS, RD
Jill Crandall, MD
Helena Duffy, MS, C-ANP
Samuel Engel, MD
Allison Friedler, BS
Crystal J. Howard-Century, MA
Stacey Kloiber, RN
Nadege Longchamp, LPN
Helen Martinez, RN, MSN, FNP-C
Dorothy Pompi, BA
Jonathan Scheindlin, MD

* denotes Principal Investigator
** denotes Program Coordinator

Elissa Violino, RD, MS
Elizabeth Walker, RN, DNSc, CDE
Judith Wylie-Rosett, EdD, RD
Elise Zimmerman, RD, MS
Joel Zonszein, MD

University of Pittsburgh **(Pittsburgh, PA)**

Trevor Orchard, MD*
Rena R. Wing, PhD*
Gaye Koenning, MS, RD**
M. Kaye Kramer, BSN, MPH**
Susan Barr, BS
Miriam Boraz
Lisa Clifford, BS
Rebecca Culyba, BS
Marlene Frazier
Ryan Gilligan, BS
Susan Harrier, MLT
Louann Harris, RN
Susan Jeffries, RN, MSN
Andrea Kriska, PhD
Qurashia Manjoo, MD
Monica Mullen, MHP, RD
Alicia Noel, BS
Amy Otto, PhD
Linda Semler, MS, RD
Cheryl F. Smith, PhD
Marie Smith, RN, BSN
Elizabeth Venditti, PhD
Valarie Weinzierl, BS
Katherine V. Williams, MD, MPH
Tara Wilson, BA

University of Hawaii **(Honolulu, HI)**

Richard F. Arakaki, MD*
Renee W. Latimer, BSN, MPH**
Narleen K. Baker-Ladao, BS
Ralph Beddow, MD
Lorna Dias, AA
Jillian Inouye, RN, PhD
Marjorie K. Mau, MD
Kathy Mikami, BS, RD
Pharis Mohideen, MD
Sharon K. Odom, RD, MPH
Raynette U. Perry, AA

Southwest American Indian Centers **(Phoenix, AZ; Shiprock, NM; Zuni, NM)**

William C. Knowler, MD, DrPH*+
Norman Cooeyate**
Mary A. Hoskin, RD, MS**
Carol A. Percy, RN, MS**
Kelly J. Acton, MD, MPH
Vickie L. Andre, RN, FNP
Rosalyn Barber
Shandiin Begay, MPH
Peter H. Bennett, MB, FRCP
Mary Beth Benson, RN, BSN
Evelyn C. Bird, RD, MPH
Brenda A. Broussard, RD, MPH, MBA, CDE
Marcella Chavez, RN, AS
Tara Dacawyma
Matthew S. Doughty, MD
Roberta Duncan, RD
Cyndy Edgerton, RD
Jacqueline M. Ghahate
Justin Glass, MD
Martia Glass, MD
Dorothy Gohdes, MD
Wendy Grant, MD
Robert L. Hanson, MD, MPH
Ellie Horse
Louise E. Ingraham, MS, RD, LN
Merry Jackson
Priscilla Jay
Roylen S. Kaskalla
David Kessler, MD
Kathleen M. Kobus, RNC-ANP
Jonathan Krakoff, MD
Catherine Manus, LPN
Sara Michaels, MD
Tina Morgan
Yolanda Nashboo (deceased)
Julie A. Nelson, RD
Steven Poirier, MD
Evette Polczynski, MD
Mike Reidy, MD
Jeanine Roumain, MD, MPH
Debra Rowse, MD
Sandra Sangster
Janet Sewenemewa
Darryl Tonemah, PhD
Charlton Wilson, MD
Michelle Yazzie

George Washington University Biostatistics Center (DPP Coordinating Center **Rockville, MD)**

Raymond Bain, PhD*
Sarah Fowler, PhD*
Tina Brenneman**
Solome Abebe
Julie Bamdad, MS
Jackie Callaghan
Sharon L. Edelstein, ScM
Yuping Gao
Kristina L. Grimes
Nisha Grover

* denotes Principal Investigator
** denotes Program Coordinator

Text S1: DPP Research Group Investigators (to Aug 2002)
Updated- June 2011

Lori Haffner, MS
Steve Jones
Tara L. Jones
Richard Katz, MD
John M. Lachin, ScD
Pamela Mucik
Robert Orlosky
James Rochon, PhD
Alla Sapozhnikova
Hanna Sherif, MS
Charlotte Stimpson
Marinella Temprosa, MS
Fredricka Walker-Murray

Central Biochemistry Laboratory **(Seattle, WA)**
Santica Marcovina, PhD, ScD*
Greg Strylewicz, PhD**
F. Alan Aldrich

Carotid Ultrasound
Dan O'Leary, MD*

CT Scan Reading Center
Elizabeth Stamm, MD*

Epidemiological Cardiology Research Center- Epicare **(Winston-Salem, NC)**
Pentti Rautaharju, MD, PhD*
Ronald J. Prineas, MD, PhD*/*
Teresa Alexander
Charles Campbell, MS
Sharon Hall
Yabing Li, MD
Margaret Mills
Nancy Pemberton, MS
Farida Rautaharju, PhD
Zhuming Zhang, MD

Nutrition Coding Center **(Columbia, SC)**
Elizabeth Mayer-Davis, PhD*
Robert R. Moran, PhD**

Quality of Well-Being Center **(La Jolla, CA)**
Ted Ganiats, MD*
Kristin David, MHP*
Andrew J. Sarkin, PhD*

NIH/NIDDK **(Bethesda, MD)**
R. Eastman, MD
Judith Fradkin, MD
Sanford Garfield, PhD

Centers for Disease Control & Prevention **(Atlanta, GA)**
Edward Gregg, PhD
Ping Zhang, PhD

University of Michigan **(Ann Arbor, MI)**
William Herman, MD, MPH

+Genetics Working Group
Jose C. Florez, MD, PhD[1, 2]
David Altshuler, MD, PhD[1, 2]
Paul I.W. de Bakker, PhD[2]
Paul W. Franks, PhD, Mphil, MS[3, 6]
Robert L. Hanson, MD, MPH[3]
Kathleen Jablonski, PhD[5]
William C. Knowler, MD, DrPH[3]
Jarred B. McAteer, AB[1, 2]
Toni I. Pollin, PhD[4]
Alan R. Shuldiner, MD[4]

1=Massachusetts General Hospital
2=Broad Institute
3=NIDDK
4=University of Maryland
5=Coordinating Center
6= Lund University, Sweden

* denotes Principal Investigator
** denotes Program Coordinator
